# Increased mutagenicity in the liver of MutaMouse females following oral treatment with commercial-grade toluene diisocyanate

**DOI:** 10.1186/s41021-025-00335-x

**Published:** 2025-07-01

**Authors:** Mariko Matsumoto, Masakatsu Natsume, Takako Iso, Takaaki Umano, Yasumasa Murata, Kenichi Masumura, Katsuyoshi Horibata, Kei-ichi Sugiyama

**Affiliations:** 1 Division of Risk Assessment, National Institute of Health Sciences, Kawasaki, Kanagawa Japan; 2 Genotoxicology Laboratory, BioSafety Research Center Inc, Iwata, Shizuoka, Japan; 3 Division of Genome Safety Science, National Institute of Health Sciences, Kawasaki, Kanagawa Japan; 4 Present address: Genotoxicology Laboratory, Trans Genic Inc, IWATA Research Institute, Iwata, Shizuoka, Japan

**Keywords:** OECD TG488, In vivo mutagenicity, Transgenic rodent gene mutation assay, Toluene diisocyanate

## Abstract

**Background:**

Toluene diisocyanates (TDIs) are high-production-volume chemicals widely used in polyurethane manufacturing. A typical commercial-grade TDI (TDI; 2,4-toluene diisocyanate: 2,6-toluene diisocyanate; 80:20), CAS: 26471-62-5, is mutagenic in *Salmonella typhimurium* with an S9 metabolic activation mix and induces chromosomal aberrations in Chinese hamster lung cells without S9 mix. While oral administration of TDI has been reported to be carcinogenic in female mice and rats of both sexes, its in vivo mutagenicity remains poorly understood. This study aimed to clarify the in vivo mutagenicity of orally administered TDI. In vivo mutagenicity was evaluated following the Organisation for Economic Co-operation and Development Test Guideline 488 (OECD TG488). MutaMouse females were orally dosed with TDI at 0 (corn oil; vehicle control), 250, 500, or 1,000 mg/kg/day for 28 days. Mutant frequencies (MFs) in the liver and glandular stomach were analyzed three days post-final dosing. Positive controls received intraperitoneal injections of *N*-ethyl-*N*-nitrosourea (ENU) at 100 mg/kg/day for two days, with MFs assessed ten days after the final dose.

**Results:**

Significant increases in *lacZ* MFs were observed in the liver at 1,000 mg/kg/day, while MFs in the glandular stomach remained unchanged. Positive controls demonstrated significantly elevated MFs in both the liver and glandular stomach.

**Conclusions:**

These findings indicate that orally administered TDI is mutagenic in mice, supporting its classification as a mutagenic carcinogen.

## Introduction

**Fig. 1:**
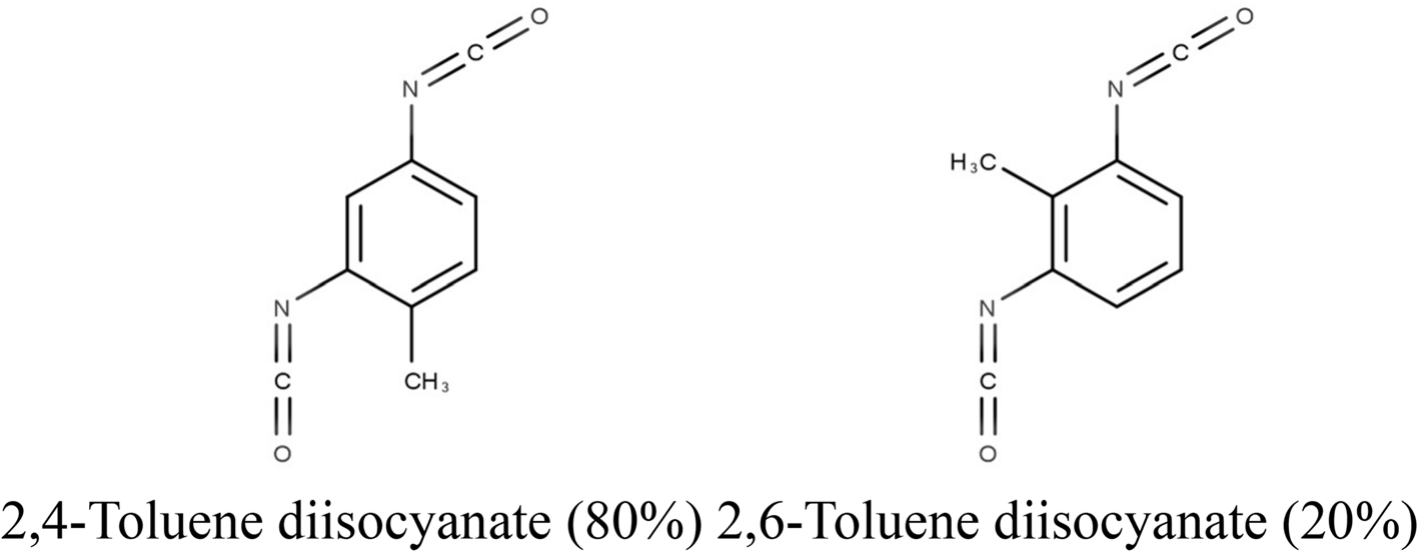
Structure of toluene diisocyanate (TDI; CAS: 26471-62-5). TDI, comprising 80% 2,4-toluene diisocyanate (2,4-TDI; CAS: 584-84-9) and 20% 2,6-toluene diisocyanate (2,6-TDI; CAS: 91-08-7)

Toluene diisocyanates (TDIs) are high-production-volume chemicals used in polyurethane production in Japan, with a total production and import volume of 87,294 t reported in 2022 [1]. TDIs are classified as priority assessment chemical substances (priority assessment No. 129) under the Chemical Substances Control Law due to their potential human health hazards. The most common isomers are 2,4-toluene diisocyanate (2,4-TDI; CAS: 584-84-9) and 2,6-toluene diisocyanate (2,6-TDI; CAS: 91-08-7). A typical commercial-grade-TDI (TDI: CAS:26471-62-5) contains 80% 2,4-TDI and 20% 2,6-TDI. The structure of TDI is shown in Fig. 1. Toxicity data for acute oral toxicity, 28-day oral repeated dose toxicity, bacterial reverse mutation, and in vitro mammalian chromosome aberration studies for TDI, conducted by the Ministry of Health, Labour and Welfare (MHLW), Japan, are publicly available in the Japan Existing Chemical Database (JECDB) [2]. Due to their extensive use and health hazard potential, many studies, including those on genotoxicity and carcinogenicity, have been conducted on TDI. The most notable adverse effect of TDI in workers is sensitization, with numerous studies reporting occupational asthma in sensitive workers [3–7]. However, sensitization is outside the scope of this study.

TDI is mutagenic in *Salmonella typhimurium* TA1538, TA98, and/or TA100 with metabolic activation [2, 8, 9]. In Chinese hamster ovary cells, 2,6-TDI induced chromosomal aberrations without S9, whereas 2,4-TDI did not induce aberrations with or without S9 [10]. TDI induced chromosomal aberrations in Chinese hamster lung cells without S9 [2]. No data exist for in vivo chromosomal aberration or micronucleus frequency following oral exposure to any isomers. Inhalation studies in rats and mice exposed to TDI for 4 weeks (6 h/day, 5 d/week) up to 0.15 ppm showed no dose- or treatment-related increases in micronucleated erythrocytes [11]. Structural chromosome aberrations, sister chromatid exchanges, and micronuclei were observed in peripheral blood lymphocytes of workers exposed to TDI (0.007–0.016 mg/m^3^), with positive results reported for all three assays [12]. However, confounding factors such as smoking and age may have contributed to these findings, as noted by Agency for Toxic Substances and Disease Registry (ATSDR, 2018) [13]. TDI-induced sex-linked recessive lethal mutations in male *Drosophila melanogaster* [14], but no mammalian studies on gene mutations were identified.

There is some evidence of carcinogenicity from TDI exposures. Rats and mice administered TDI in corn oil by gavage at 30–120 mg/kg/day (rats) or 60–240 mg/kg/day (mice) for 105 or 106 weeks (5 d/week) developed subcutaneous tissue fibromas and fibrosarcomas in male and female rats, pancreatic acinar cell adenomas in male rats, pancreatic islet cell adenomas, neoplastic nodules of the liver, mammary gland fibroadenomas in female rats and hemangiomas, hemangiosarcomas, and/or hepatocellular adenomas in female mice [15]. Unlike female mice, male mice did not exhibit carcinogenic effects under these study conditions. Long-term inhalation toxicity study, rats and mice exposed to TDI at 0.05 and 0.15 ppm (6 h/day, 5 d/week) for approximately 2 years did not show any carcinogenic effects [11]. No human carcinogenicity studies for oral administration have been reported. The risk of cancer associated with inhalation exposure to TDI at polyurethane foam manufacturing facilities has been evaluated in three industrial cohort studies [16–21]. While some studies suggested an association between occupational exposure and lung cancer in female workers [17–19], no strong association or consistent pattern was observed when considering confounding factors. Based on sufficient evidence in experimental animals and inadequate evidence in humans, TDI is classified by IARC as possibly carcinogenic to humans (Group 2B) [22].

TDI is mutagenic in vitro, and orally administered TDI is carcinogenic in laboratory animals. However, its in vivo mutagenicity has not been well studied. Therefore, this study aimed to clarify the in vivo mutagenicity of orally administered TDI using transgenic MutaMouse. Our study will be useful to understand whether TDI is a mutagenic carcinogen or not.

## Materials and methods

This experiment was  conducted at the BioSafety Research Center (BSRC: Shizuoka, Japan) following the OECD Guidelines for Chemical Testing 488 (OECD TG 488). Animal experiments were conducted in accordance with the regulations of the Animal Care and Use Committees of the National Institute of Health Sciences (approval protocol No. 870). It was approved under the BSRC Safety Management Regulations for Recombinant DNA Experiments. The study adhered to (1) the Act on Animal Welfare and Management (Act No. 105), (2) standards relating to the care and management of laboratory animals and pain relief (Japan Ministry of the Environment Notice No. 88), (3) BSRC Guidelines for Animal Experimentation, and (4) the Act on the Conservation and Sustainable Use of Biological Diversity through Regulations on the Use of Living Modified Organisms (Act No. 97).

### Chemicals

Toluene diisocyanate (TDI; CAS: 26471-62-5, Lot No. LEE6335, purity: 97.2%) and corn oil (vehicle) were purchased from FUJIFILM Wako Pure Chemical Corporation (Osaka, Japan). TDI consists of 80% 2,4-toluene diisocyanate (CAS: 584-84-9) and 20% 2,6-toluene diisocyanate (CAS: 91-08-7). *N*-ethyl-*N*-nitrosourea (ENU), the positive control substance, was obtained from Toronto Research Chemicals Inc. (Ontario, Canada).

### Animals and treatment

Male and female CD2 F1/Slc mice for the dose-finding study were procured from Japan SLC, Inc. (Shizuoka, Japan). Female CD_2_-LacZ80/HazfBR (MutaMouse) for the main study were sourced from BSRC (Kobe, Japan). After acclimation, animals aged 9 weeks and in good health were selected for the study. They were provided a basal diet, CRF-1 (Oriental Yeast), and water *ad libitum*. Animals were maintained at a temperature of 20 °C to 26 °C, relative humidity of 35–70%, a 12 h light/dark cycle, and 12 air changes per hour.

### Dose-finding study

In the dose-finding study, CD2 F1/Slc mice (3/sex) received 30, 100, 300, or 1,000 mg/kg/day by oral gavage in a treatment volume of 10 mL/kg once daily for 14 days. We selected CD2 F1/SLC mice because they are used as the non-transgenic animals of same rodent strain of in vivo gene mutation assay. Body weight was recorded on Days 1, 8, and 15, with daily monitoring for mortality and clinical signs. Surviving animals were euthanized using carbon dioxide gas on Day 15. No mortality or treatment-related body weight changes were observed at doses up to 1,000 mg/kg/day. Clinical signs of toxicity at 1,000 mg/kg/day included ulcers in the anogenital or lumbar regions and watery feces. No sex-specific differences in toxicity were noted, but females were selected for the main study due to carcinogenic effects reported in female mice, as described in the introduction. The main study dosages were set at 0, 250, 500, and 1,000 mg/kg/day, with 1,000 mg/kg/day being the limit dose recommended by OECD TG488.

### Main study

Four groups of female MutaMouse were administered 0 (vehicle: corn oil), 250, 500, and 1,000 mg/kg/day at a constant volume of 10 mL/kg once daily for 28 days. Six or eight animals (at 1,000 mg/kg/day) per group were treated to ensure five animals per group for analysis. Positive control animals were treated with ENU intraperitoneally (i.p.) at 100 mg/kg/day once daily for 2 days. Euthanasia of experimental animals and extraction of target organs were performed 3 days after the final administration for treatment and vehicle control groups and 10 days after for the positive control group. The liver and glandular stomach were collected post-euthanasia using carbon dioxide gas, and a gross pathology examination was conducted. All animals were observed daily, and body weight was recorded on Days 1, 8, 15, 22, 29, and 31 for the vehicle control and treatment groups, and on Days 1, 8, and 20 for the positive control group.

For mutation analysis, we selected the animals to sequence based on the animal ID numbers, from lowest to highest in every dose group. In the first study, *N* = 5 was used for all dose groups. Although a statistically significant increase in mutant frequency was observed in the liver at 1,000 mg/kg/day, no clear dose-dependency was confirmed, leading to the selection of all liver samples for reanalysis (the second study). The second study used *N* = 6 for control, low and middle dose, and *N* = 8 for the high dose group. For the glandular stomach, *N* = 5 was used for all dose groups.

Two sections of the left lateral lobe of the liver were excised and frozen in liquid nitrogen in individual microtubes. The remaining parts and lobes were placed in a storage bag and frozen while being compressed with a flat-bottom metal container filled with liquid nitrogen. The glandular stomach was frozen in liquid nitrogen within a laboratory storage bag. All frozen samples were stored at − 80 °C until analysis.

### DNA isolation

Genomic DNA was isolated from the liver and glandular stomach of five animals per group in ascending order of animal ID. Given the statistically significant increase in mutant frequency in the liver at 1,000 mg/kg/day without clear dose-dependency, additional DNA extractions were conducted on all liver samples for further evaluation. The method for genomic DNA isolation has been previously described [23]. Briefly, homogenized frozen tissues in Dounce buffer were transferred to ice-cold centrifuge tubes containing sucrose. After centrifugation at 3,000 rpm (1,750G) for 10 min, the supernatant was removed. Precipitated nuclei/cells were suspended in Dounce buffer containing RNase (NIPPON GENE Co., Ltd.), mixed with proteinase K solution (FUJIFILM Wako Pure Chemical Co., Ltd.), and digested at 50 °C for 2.5 h. The suspension was mixed with phenol/chloroform solution, rotated for 10 min, and centrifuged at 2,500 rpm (1,220G) for 10 min. The aqueous layer was mixed with chloroform/isoamyl alcohol solution and extracted similarly. Genomic DNA was precipitated with ethanol, air-dried, and dissolved in TE buffer (NIPPON GENE Co., Ltd.). The ratio of absorbance at 260 nm and 230 nm was confirmed to be 1.7 or more. DNA concentration (241~622 µg/mL) was measured using a NanoDrop (AGC TECHNO GLASS Co., Ltd.).

### In vitro packaging

The packaging reaction for transgene rescue was performed following the Transpack instruction manual (Agilent Technologies). Approximately 10 µL of the genomic DNA solution was gently mixed with the Transpack packaging tube and incubated at 30 °C for 90 min, repeated twice, before being combined with 700 µL of SM buffer.

### Mutant frequency determination

A suspension of *Escherichia coli* C strain (*lacZ−*,* galE−*) was mixed with the entire packaged sample (~ 700 µL volume), stirred, and incubated for 30 min to allow the rescued phages absorb into the *E. coli*. This solution was diluted 10-fold by adding 30-µL of the sample to 270 µL of LB culture medium containing 10 mmol/L magnesium sulfate. Subsequently, 30 µL of this dilution was mixed with LB top agar for the titer plates. While the remaining cell suspension was mixed with LB top agar containing P-gal (phenyl-β-D-galactoside) for the selection plates. These plates were incubated overnight at 37 °C. The packaging procedures were repeated until a total of 300,000 plaques were produced.

The total number of plaques on the titer plate (N) was counted, and the total number of plaques was calculated using the formula: (*N* × 300 µL × 2,700 µL)/(30 µL × 30 µL) = 900 N.

The mutant frequency was determined as the total number of mutant plaques on selection plates divided by the total number of rescued plaques.

### Statistical analysis

For multiple comparisons between the vehicle control group and the treatment groups, Bartlett’s test was used to assess the homogeneity of MFs in the treatment and vehicle control groups. Dunnett’s test was applied for homogeneous data, while Steel’s test was used for non-homogenous data. For a comparison between two groups, vehicle and positive controls, either Student’s *t*-test or Aspin-Welch’s *t*-test was employed to evaluate MF differences, depending on the F-test outcome. The significance criterion was set at 5%. The Cochran-Armitage test was used for trend analysis. A *p*-value less than 0.05 was judged to be a significant trend in the data.

## Results

### Clinical signs of toxicity, body weight, and gross pathological examination

**Fig. 2:**
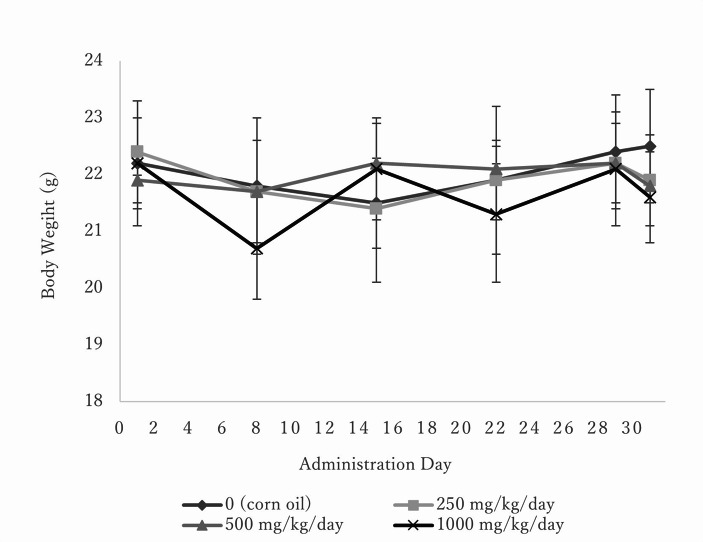
Body weights of female transgenic mice gavaged with toluene diisocyanate (TDI) for 28 days. *N* = 6 for 0, 250 and 500 mg/kg/day, and *N* = 8 for 1000 mg/kg/day

In the main study, no clinical signs of toxicity were observed at 250 or 1,000 mg/kg/day. Watery feces were noted in one animal at 500 mg/kg/day. Body weights were unaffected at any dose during the administration period (Fig. 2). Gross pathological examination revealed no dose-related effects (data not shown).

### Mutation assay

#### LacZ MFs in the liver

Two experiments were done to quantify mutant frequency in the liver samples. In the first experiment with *N* = 5, the MFs (mean value ± SD) were 44.2 ± 10.6 (×10^–6^), 92.1 ± 76.4 (×10^–6^), 69.8 ± 32.4 (×10^–6^), and 98.1 ± 31.1 (×10^–6^) at 0 (vehicle control), 250, 500, and 1,000 mg/kg/day, respectively (Table 1). A significant difference was observed in the 1,000 mg/kg/day group, with *p*-values (Steel’s test) of 0.6768, 0.1216, and 0.0251 for the 250, 500, and 1,000 mg/kg/day groups, respectively, compared to the vehicle control group. The MF in the positive control group was statistically significantly increased (Aspin-Welch’s *t-*test) compared to the vehicle control group. Table 2 presents the results of an additional assay for the livers. In the second experiment with *N* = 6 to 8, the MFs were 40.5 ± 10.3 (×10^–6^), 90.8 ± 58.7 (×10^–6^), 70.6 ± 15.3 (×10^–6^), and 159.0 ± 121.0 (×10^–6^) at 0 (vehicle control), 250, 500, and 1,000 mg/kg/day, respectively. Statistically significant differences were noted in the 500 and 1,000 mg/kg/day groups compared to the vehicle control group, with *p*-values of 0.0659, 0.0285, and 0.0056 for the 250, 500, and 1,000 mg/kg/day groups, respectively. The fold changes were 2.2, 1.7, and 3.9 at 250, 500, and 1,000 mg/kg/day, respectively. As expected, the positive control produced statistically significant results. A significant increasing trend of the MF was confirmed (*p* < 0.001) for both assays.

**Table 1:** The *lacZ* mutant frequencies (MF) in the liver of female transgenic mice gavaged with toluene diisocyanate (TDI) for 28 days, evaluated 3 days after the final administration (first assay)

Substance	Dose (mg/kg/day)	Animal ID No.	No. of rescued phages	No. of mutants	MF (×10^−6^)	Mean	±	S.D. (×10^−6^)		Fold change
Corn oil	0	3001	843,300	46	54.5					
		3002	947,700	49	51.7					
		3003	607,500	22	36.2	44.2	±	10.6		
		3004	702,000	21	29.9					
		3005	432,900	21	48.5					
TDI	250	3101	387,000	7	18.1					
		3102	610,200	28	45.9					
		3103	503,100	48	95.4	92.1	±	76.4		2.1
		3104	437,400	95	217.2					
		3105	405,900	34	83.8					
	500	3201	482,400	28	58.0					
		3202	587,700	74	125.9					
		3203	363,600	23	63.3	69.8	±	32.4		1.6
		3204	402,300	17	42.3					
		3205	318,600	19	59.6					
	1,000	3301	528,300	47	89.0					
		3302	590,400	84	142.3					
		3303	541,800	35	64.6	98.1	±	31.1	*(S), ^#^	2.2
		3304	496,800	39	78.5					
		3305	301,500	35	116.1					
ENU	100	3401	717,300	82	114.3					
		3402	873,000	159	182.1					
		3403	397,800	112	281.5	177.0	±	64.5	*(AW)	4.0
		3404	355,500	61	171.6					
		3405	546,300	74	135.5					

Corn oil: Vehicle control (10 mL/kg)

ENU: Positive control (*N*-ethyl-*N*-nitrosourea, 10 mL/kg, i.p., dose once a day, for 2 days, expression period; 10 days)

*Significant difference from vehicle control (*p* < 0.05) (S): Steel’s test (AW): Aspin-Welch’s *t*-test

^#^Significant increase trend (*p* < 0.001) by the Cochran-Armitage test

**Table 2:** The *lacZ* mutant frequencies (MF) in the liver of female transgenic mice gavaged with toluene diisocyanate (TDI) for 28 days, evaluated 3 days after the final administration (additional assay)

Substance	Dose (mg/kg/day)	Animal ID No.	No. of rescued phages	No. of mutants	MF (×10^−6^)	Mean	±	S.D. (×10^−6^)		Fold change
Corn oil	0	3001	603,900	15	24.8					
		3002	963,000	36	37.4					
		3003	650,700	23	35.3	40.5	±	10.3		
		3004	692,100	31	44.8					
		3005	433,800	20	46.1					
		3006	603,000	33	54.7					
TDI	250	3101	456,300	21	46.0					
		3102	564,300	29	51.4					
		3103	1,193,400	179	150.0	90.8	±	58.7		2.2
		3104	371,700	67	180.3					
		3105	306,000	16	52.3					
		3106	403,200	26	64.5					
	500	3201	491,400	24	48.8					
		3202	648,000	35	54.0					
		3203	374,400	30	80.1	70.6	±	15.3	*(S)	1.7
		3204	577,800	50	86.5					
		3205	366,300	28	76.4					
		3206	567,900	44	77.5					
	1,000	3301	632,700	73	115.4					
		3302	459,000	56	122.0					
		3303	429,300	90	209.6	159.0	±	121.0	*(S), ^#^	3.9
		3304	409,500	34	83.0					
		3305	719,100	60	83.4					
		3306	412,200	182	441.5					
		3307	311,400	30	96.3					
		3308	662,400	80	120.8					
ENU	100	3401	741,600	67	90.3					
		3402	384,300	66	171.7					
		3403	787,500	210	266.7	175.6	±	75.2	*(AW)	4.3
		3404	617,400	118	191.1					
		3405	301,500	74	245.4					
		3406	531,000	47	88.5					

Corn oil: Vehicle control (10 mL/kg)

ENU: Positive control (*N*-ethyl-*N*-nitrosourea, 10 mL/kg, i.p., dose once a day, for 2 days, expression period; 10 days)

*Significant difference from vehicle control (*p* < 0.05) (S): Steel’s test (AW): Aspin-Welch’s *t*-test

^#^Significant increase trend (*p* < 0.001) by the Cochran-Armitage test

#### LacZ MFs in the glandular stomach

The results for MFs in the glandular stomach are shown in Table 3. The mean MFs were 39.1 ± 7.6 (×10^–6^), 46.8 ± 15.7 (×10^–6^), 43.0 ± 2.5 (×10^–6^), and 47.5 ± 17.8 (×10^–6^) at 0 (vehicle control), 250, 500, and 1,000 mg/kg/day, respectively. No statistically significant differences were observed between the treatment groups and the vehicle control group. The MF in the positive control group was statistically significantly increased (Aspin-Welch’s *t* t-test) compared to the vehicle control group.

**Table 3:** The *lacZ* mutant frequencies (MF) in the glandular stomach of female transgenic mice gavaged with toluene diisocyanate (TDI) for 28 days, evaluated 3 days after the final administration

Substance	Dose (mg/kg/day)	Animal ID No.	No. of rescued phages	No. of mutants	MF (×10^−6^)	Mean	±	S.D.(×10^−6^)		Fold change
Corn oil	0	3001	479,700	20	41.7					
		3002	856,800	32	37.3					
		3003	889,200	30	33.7	39.1	±	7.6		
		3004	942,300	30	31.8					
		3005	373,500	19	50.9					
TDI	250	3101	673,200	22	32.7					
		3102	538,200	20	37.2					
		3103	567,000	21	37.0	46.8	±	15.7		1.2
		3104	486,000	33	67.9					
		3105	489,600	29	59.2					
	500	3201	648,000	26	40.1					
		3202	836,100	36	43.1					
		3203	909,900	41	45.1	43.0	±	2.5		1.1
		3204	586,800	24	40.9					
		3205	851,400	39	45.8					
	1,000	3301	453,600	17	37.5					
		3302	639,900	48	75.0					
		3303	652,500	36	55.2	47.5	±	17.8		1.2
		3304	592,200	23	38.8					
		3305	516,600	16	31.0					
ENU	100	3401	664,200	241	362.8					
		3402	396,000	151	381.3					
		3403	383,400	174	453.8	403.6	±	35.3	*(AW)	10.3
		3404	506,700	213	420.4					
		3405	385,200	154	399.8					

Corn oil: Vehicle control (10 mL/kg)

ENU: Positive control (*N*-ethyl-*N*-nitrosourea, 10 mL/kg, i.p., dose once a day, for 2 days, expression period; 10 days)

*Significant difference from vehicle control (*p* < 0.05) (AW): Aspin-Welch’s *t*-test

## Discussion

The MFs of the liver and glandular stomach in female MutaMouse gavaged with TDI for 28 days were evaluated. The MFs were significantly increased in the liver but not in the glandular stomach. These findings suggest that orally administered TDI is mutagenic in mice, and TDI is considered a mutagenic carcinogen.

The mean MF in the liver was significantly increased at 1,000 mg/kg/day in the first assay. However, the mean MF was higher in the 250 mg/kg/day group (92.1 ± 76.4 (×10^–6^)) than in the 500 mg/kg/day group (69.8 ± 32.4 (×10^–6^)). Although a significant increasing trend was confirmed, dose dependency was unclear with large SDs in this assay. In the 250 mg/kg/day group, individual MFs varied widely, with values ranging from 18.1 (×10^–6^) to 217.2 (×10^–6^). Historical control data of the mean MFs (mean value ± SD) for the vehicle and positive controls in the liver of female MutaMouse (*N* = 20) at the facility are 47.8 ± 10.2 (×10^–6^) and 150.8 ± 39.3 (×10^–6^), respectively. The SDs for the first liver assay were large compared to the historical control data.

The second assay using 6 or 8 animals per group was conducted for the liver to confirm dose dependency. In the second assay, the mean MFs in the livers of MutaMouse given TDI were significantly increased at 500 and 1,000 mg/kg/day. Two animals (Animal IDs 3103 and 3104) in the 250 mg/kg/day group showed high MFs of 150.0 (×10^–6^) and 180.3 (×10^–6^), resulting in a higher mean MF at 250 mg/kg/day compared to 500 mg/kg/day. A significant increasing trend was confirmed, and dose-dependency for the MFs was confirmed in the second liver assay. However, the SDs were still large. The MFs appeared to be less reproducible between two assays for some individuals (e.g., Animal IDs 3202 and 3303). The two assays were performed using the liver DNA (1 st and 2nd extraction) prepared from the same individual and examining almost equal number of plaques. On the other hand, each DNA extraction used a different piece of the same frozen tissue; therefore, the different DNA preparations from the same individual may have impacted the variation of liver MF.

The liver MFs of two animals in 250 mg/kg/day (Animal IDs 3103 and 3104) were relatively high within the group with reproducibility. This result may suggest that the clonally expanded mutations may occur and affect the higher MFs in some individuals. It may be necessary to sequence the *lacZ* gene, if possible, to confirm the presence of clonal mutations.

The large variation of individual MF in the liver might also be related to a unique phenomenon to oral gavage dose of TDI. At low concentrations, 2,6- and 2,4-TDIs hydrolyze to 2,6- and 2,4-diaminotoluenes (2,6-DAT and 2,4-DAT), respectively, but at high concentrations, they form oligo-/poly-ureas through the reaction of amino groups with the isocyanate [24, 25]. The percentage yields of 2,4-DAT from 2,4-TDI with water at 27 °C were reported as 61%, 19%, 2%, and 0.4% at concentrations of 10, 100, 1,000, and 10,000 mg/L, respectively [25]. The structures of the dominant products of 2,4-TDI reactions are shown in Table 4.

**Table 4:** Dominant metabolite or reaction product of 2,4-toluene diisocyanates (TDI) (as reported by Timchalk et al., [26])

Route of exposure	Dominant metabolite/reaction product
Oral (low concentration)	Hydrolyzed to 2,4-DAT in the gastrointestinal tract
Oral (high concentration)	Forming TDI oligo-/poly-urea
Inhalation	Forming adducts with macromolecules

Both 2,4-DAT and 2,6-DAT have been shown to be mutagenic in bacterial reverse mutation assays with S9 mix. Additionally, male F344 *gpt* delta rats orally administered 2,4-DAT or 2,6-DAT for 28 days or 13 weeks developed positive foci in the liver when treated with 2,4-DAT but not with 2,6-DAT [27, 28]. These findings suggest that 2,4-DAT, hydrolyzed from 2,4-TDI (comprising 80% of TDI), is responsible for the observed positive mutagenicity in the liver. The large individual differences in MFs and the reversed MFs between 250 mg/kg/day and 500 mg/kg/day in this study can be attributed to the varying amounts of 2,4-DAT hydrolyzed in the gastrointestinal tracts of individual animals. For example, the state of TDI in the corn oil vehicle could influence the degree of hydrolyzation. In this study, the mean MFs in the glandular stomach were not increased up to 1,000 mg/kg/day. This result further suggests that 2,4-DAT may undergo additional metabolism to mutagenic compounds in the liver.

As previously noted, there were no dose-related increases in micronucleated erythrocytes in rats and mice exposed to TDI via inhalation for 4 weeks (6 h/day, 5 d/week) at 0.15 ppm [11]. The authors of this study also confirmed the absence of carcinogenic findings in rats and mice following 2 years of inhalation exposure [11]. According to Timchalk et al. (1994), the metabolic pathway of TDI differs between oral and inhalation exposures. After inhalation, 2,4-TDI conjugates with macromolecules such as hemoglobin, albumin, glutathione, and others, and is primarily excreted via feces (Table 4) [26]. Schupp and Plehiers (2022) reviewed four decades of literature on the absorption, distribution, metabolism, and excretion of isocyanate groups. They indicated that isocyanate groups are scavenged at the portal of entry and are not systemically available in an unbound reactive form [29]. This finding could explain why inhalation exposure to TDI does not induce genotoxicity or carcinogenicity in laboratory animals. The most likely route of human exposure to TDI is inhalation. While carcinogenicity was not observed in animal inhalation studies, and no strong association or consistent pattern of carcinogenicity was found in occupational exposure studies, whether 2,4-DAT is produced in the local respiratory tract after TDI inhalation requires further investigation.

TDI administered via gavage is carcinogenic in rats of both sexes and in female mice, but not in male mice [15]. Similarly, dietary 2,4-DAT increased the incidence of hepatic neoplasia in male and female rats and mammary tumors in female rats [30, 31], while it induced hepatic neoplasia only in female mice [31]. The current study confirms that orally administered TDI induces mutations in the livers of female mice, suggesting that TDI is a mutagenic carcinogen. Species differences between rats and mice in the carcinogenic potential of 2,4-DAT have been previously discussed [32, 33]. These differences may relate to the higher levels of *N*-acetyltransferase in mice, which produce less toxic *N*-acetylated metabolites. Greater levels of 4-acetylamino-2-aminotoluene and 2,4-diacetylaminotoluene, metabolites of 2,4-DAT, in rats appear to contribute to the formation of carcinogenic aromatic amines and amides [33]. Genetic variants of *N*-acetyltransferase genes are associated with diisocyanate asthma in diisocyanate-exposed workers [34]. Such genetic variations may also influence susceptibility to TDI-induced carcinogenicity in humans. However, female mice exhibit higher *N*-acetyltransferase activity for 2,4-DAT than males [32]; thus, the mechanism underlying the sex differences in TDI carcinogenicity requires further research.

## Conclusions

We used OECD TG488 to clarify the in vivo mutagenicity of orally administered TDI in female mice. The MFs were significantly increased in the liver but not stomach. We conclude that TDI should be considered a mutagenic carcinogen. Our findings will be useful for the risk assessment of TDI.

## Data Availability

No datasets were generated or analysed during the current study.
